# Enzymatic Synthesis of Functional PEGylated Adipate Copolymers

**DOI:** 10.1002/cplu.202400668

**Published:** 2025-03-12

**Authors:** Eleni Axioti, Emily G. Dixon, Thomas Jepras, Fen Tin He, Peter J. V. Hartman, Bradley Hopkins, Vincenzo Di Bari, Jiraphong Suksiriworapong, Valentina Cuzzucoli Crucitti, Luciano Galantini, Iolanda Francolini, Robert J Cavanagh, Vincenzo Taresco

**Affiliations:** ^1^ School of Chemistry University Park Nottingham NG7 2RD United Kingdom; ^2^ Division of Food Nutrition and Dietetics School of Biosciences University of Nottingham Sutton Bonington Campus Nottingham LE12 5RD United Kingdom; ^3^ Department of Pharmacy Faculty of Pharmacy Mahidol University Bangkok 10400 Thailand; ^4^ Centre for Additive Manufacturing Department of Chemical and Environmental Engineering University of Nottingham University Park Nottingham NG7 2RD United Kingdom; ^5^ Department of Chemistry Sapienza University of Rome Piazzale A. Moro 5 Rome 00185 Italy; ^6^ School of Pharmacy University Park Nottingham NG7 2RD United Kingdom

**Keywords:** Enzymatic polymerization, Polycondensation, PEG, Nanoparticles, Drug delivery

## Abstract

Many new active pharmaceutical ingredients (APIs) demonstrate high hydrophobicity and low water‐solubility issues. In this regard, polymeric nanoparticles (NPs) have been extensively used as drug delivery carriers for the encapsulation of such APIs. One commonly used polymer is polyethylene glycol (PEG), owing to its biocompatibility, high water solubility, and capacity to prolong the drug residence time. However, concerns have arisen regarding PEG's immunogenicity and limited biodegradability. In addition, inherent limitations, including limited chemical handles can restrict PEG's effectiveness in physiological conditions. For this reason, in the present study, we combine the advantages offered by PEG with the use of an enzymatic synthetic route to produce novel PEGylated polyesters. Furthermore, it has been proven that incorporation of hydrophobic diols into the PEGylated backbone influences NPs formation, stability, and drug encapsulation, despite high chemical similarity. As a preliminary result, samples containing PEG and 1,6‐hexanediol in a 50 : 50 ratio (PEGA‐Hex 50 %) and PEG and 2‐hydroxyethyl disulfide in a 50 : 50 ratio (PEGA‐SS 50 %) have proved to be the most promising candidates in this small library analysed. Both samples exhibited sufficient NPs stability, biocompatibility, and superior encapsulation efficiency compared to the other variants.

## Graphical abstract

This study explores a one‐pot, one‐step enzymatic polycondensation process to create amphiphilic PEG‐adipate variants with improved physicochemical properties that can self‐assemble into nanoparticles. By varying hydrophobic and functionalised diols, the impact on nanoparticle formation, stability, and drug encapsulation is examined. The results highlight the potential of these PEGylated polymers for sustainable nano‐drug delivery systems.

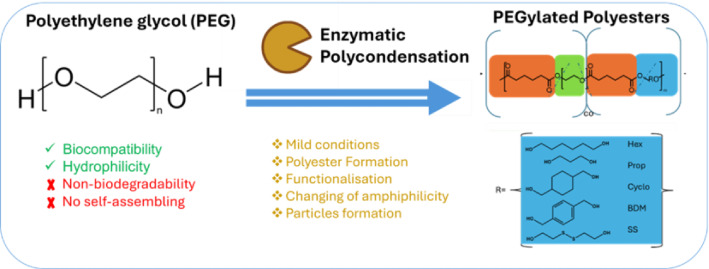

## Introduction

Drug delivery systems are under continuous development to provide more effective ways to deliver drugs.[^1^, ^2^] However, different drugs have different physicochemical and biological properties, necessitating different formulation strategies.^[3]^ Either poor drug solubility or permeability is a rate‐limiting step in determining bioavailability after drug administration. Enhancing drug solubility and permeability has consistently posed a challenge in optimising bioavailability and remains a key priority in pharmaceutical research.[^2^, ^4^] In recent years, many advancements have been made in drug delivery systems (DDS) to address this issue, with polymeric systems being used extensively for entrapping molecules, and therefore improving water solubility and absorption.[^4^, ^5^, ^6^] In this context, polymeric nanoparticles (NPs) comprise amphiphilic polymers that can self‐assemble in aqueous media and encapsulate hydrophobic drug molecules within their hydrophobic core, providing a mechanism for drug dissolution.[^7^, ^8^, ^9^]

To date, one of the most widely used polymers in clinical formulations is the semicrystalline polyether polyethylene glycol (PEG).^[10]^ PEG is generally used in its linear architecture and can conjugate to other polymers or small molecules only via end groups (usually one or two hydroxyl groups).^[11]^ Furthermore, PEG is often the clinical polymer of choice due to its biocompatibility.[^12^, ^13^] Owing to its high polarity and hydrophilic nature,^[14]^ PEGs at different molar masses are readily soluble in water and electrically neutral throughout the entire pH range.^[15]^ Due to these characteristics, PEG can either physically adhere to NPs by physical adsorption, including electrostatic or hydrophobic interactions, or be directly attached to the surface of NPs covalently.^[11]^ However, the most common strategy is for PEG to be covalently attached, as the hydrophilic moiety, to a hydrophobic polymeric chain, creating an amphiphilic block copolymer.[^10^, ^11^, ^16^] The latter, *PEGylation* describes the conjugation of PEG with synthetic polymers, biomolecules, and nanoparticles *via* its functional groups.^[14]^ PEGylation with hydrophobic moieties affects amphiphilicity, thereby facilitating self‐assembly into NPs with simultaneous encapsulation of hydrophobic drugs, enhancing their aqueous solubility, as well as physical and thermal stability.[^10^, ^16^, ^17^] Combining encapsulation and PEGylation can lead to improved delivery of drugs to specific sites of action in the body, reducing opsonisation, henceforth providing a prolonged residence of the drug *in vivo*, a decreased degradation by metabolic enzymes, and a reduction or elimination of protein immunogenicity.[^18^, ^19^] Furthermore, PEG reduces the tendency of particles to aggregate by steric stabilization, thereby producing formulations with increased stability during storage and application.^[19]^

Despite the many advantages of PEGylation, concerns have been raised due to its limited biodegradation, tissue accumulation, and potential for immunogenicity.[^14^, ^20^, ^21^] Adverse side effects in the body, provoked by PEG, may lead to hypersensitivity, faster drug clearance, and occasionally life‐threatening side effects.^[22]^

Synthetic aliphatic polyesters are widely used for biomedical, pharmaceutical, and environmental applications due to their high biodegradability, biocompatibility, and low cost of production.^[23]^ Furthermore, the physiochemical properties of polyesters are *tuneable ‐* crystallinity, thermal transitions, mechanical strength, and degradation can be altered based on molar mass, composition, or addition of substituents to the polyester backbone.^[24]^ Biodegradable polyesters show significant potential as drug delivery carriers, undergoing biodegradation *in vivo*, yielding biocompatible or non‐toxic by‐products, and offering an overall reduction of side effects.^[25]^ When PEGylated, they have been used successfully to form stable and *stealth* nanoparticles for drug delivery applications.[^24^, ^26^, ^27^] Such polyesters can be synthesised either via ring‐opening polymerisation (ROP) or polycondensation (PC) reactions using a wide range of catalysts such as organic bases and metal‐based organocatalysts.[^28^, ^29^] Contrastingly, enzymatic polymerisation can offer a greener synthetic alternative for the production of biodegradable polymers, including aliphatic polyesters, typically synthesised by lipases.[^30^, ^31^, ^32^] As polymerisation catalysts, enzymes have several characteristic features when compared to conventional chemical catalysts concerning regio‐ and stereo‐selectivity, high catalytic activity, lack of undesirable side reactions, and operation under mild conditions.[^33^, ^34^]

As a poorly water‐soluble model drug, Curcumin, a natural bioactive compound extracted from the rhizomes of the genus *Curcuma*, has gained significant attention for its potential in developing nutraceutical, cosmeceutical, and pharmaceutical products. Such widespread use is a consequence of its diverse biological activities, including antioxidant, anti‐inflammatory, anticancer, cardioprotective, and neuroprotective effects.^[35]^ However, curcumin, a hydrophobic molecule with limited gastrointestinal absorption upon oral administration, encounters significant bioavailability issues. Despite these limitations, curcumin has been shown to remain chemically stable in the stomach and intestines for a sufficient duration allowing for potential absorption if solubilized.^[36]^ Consequently, various strategies aimed at enhancing curcumin's aqueous solubility and permeability hold promise for developing more effective curcumin formulations.[^37^, ^38^] Additionally, Coumarin 6 (Coum6) is a fluorescent, water‐insoluble small molecule (dye) that has previously been used as model drug in encapsulation studies[^39^, ^40^] and is suitable for imaging experiments as a model drug, as also demonstrated in this study.[^41^, ^42^]

The present study aims to improve the use of PEG by developing PEGylated polyesters with surfactant properties, allowing for favourable interactions with both hydrophobic drugs and aqueous media. The ester bonds not only deliver amphiphilic balance but also provide a potential mechanism for hydrolysis *in vivo* ^[14]^ or enzymatic degradation.[^23^, ^39^, ^40^] The chosen activated ester monomer for this polymer is divinyl adipate (DVA), which has been enzymatically polymerised with PEG400 to form the amphiphilic copolymer polyethylene glycol adipate (PEGA). By selecting a small molar mass PEG unit, the aim is to generate short chains that can be excreted from the body, owing to low molar mass below the renal clearance threshold.^[43]^ Furthermore, the addition of a range of new functionalities offered by different diols, including i)1,4‐hexanediol, ii) 1,3‐propanediol, iii) 1,4‐cyclohexanedimethanol, iv) 1,4‐benzenedimethanol and v) 2‐hydroxyethyl disulfide, have been used to compare and screen physical properties, self‐assembling ability, biostability and finally drug encapsulation efficiency.

## Materials and Methods

### Materials

Divinyl adipate (DVA) was purchased from Tokyo Chemical Industries, UK. PEG (400),1,6‐hexanediol (Hex), 1,3‐propanediol, and 2‐hydroxyethyl disulfide were purchased from Sigma‐Aldrich, UK. Phosphate buffer saline (PBS), bovine serum albumin (BSA), Coumarin 6, Curcumin, Novozyme 435 lipase, derived from Candida antarctica immobilised on an acrylic macroporous resin, and Lipase from porcine pancreas were also purchased from Sigma‐Aldrich. 1,4‐Cyclohexanedimethanol was purchased from Thermo Scientific and 1,4‐benzenedimethanol from Acros Organics. Solvents were purchased from Fisher Scientific UK (Tetrahydrofuran) and Sigma‐Aldrich (Acetone, Acetone d6). All chemicals and solvents were used as received without further purification.

### Methods

#### Nuclear Magnetic Resonance Spectroscopy (NMR)

Successful polymer synthesis was confirmed using 1H NMR spectroscopy. NMR spectra were recorded using a Bruker DPX 400 MHz spectrometer (Germany) using acetone‐d6 solvent. Chemical shifts are given in ppm. Approximately 5 mg of polymer was dissolved in 0.6–0.7 mL of solvent. MestReNova 14.3.2 copyright 2023 (Mestrelab Research S.L.) was used for analysis.

#### Gel Permeation Chromatography (GPC)

Polymer Number Average Molar Mass (Mn) and dispersity (*Ð*) were determined using a GPC systems of THF (HPLC grade) eluent at 40 °C. Chromatographs were recorded using two Agilent PL‐gel mixed D columns for THF system in series with a flow rate of 1 mL min^−1^ and an injection loop of 50 μL. Samples were detected using a differential refractometer (DRI). Samples were prepared by dissolving the samples (5 mg) in THF (2 mL) and then filtering all samples through 0.22 μm Teflon filter. Low dispersity (*Ð*) poly (methyl methacrylate) standards were used for the system calibration with average molar masses ranging from 540 to 1.02×106 g mol^−1^.

#### Differential Scanning Calorimetry (DSC)

Thermal properties of the polymers were determined using DSC. Analysis was performed on a TA‐Q2000 (TA instruments), calibrated with sapphire and indium standards under N2 flow at 50 mL min^−1^. Polymer (∼5 mg) was weighted into a T‐zero aluminium pan (TA instruments) with a reference pan (T‐zero aluminium) remaining empty. Pan lids were pin‐holed, due to the tackiness of the polymer, and samples were heated and cooled at a rate of 10 °C min from −90 to 200 °C. Two heating cycles were recorded in order to remove any thermal history of the polymers. The second heating cycle was used to determine the glass transition temperature (T_g_) and melting temperature (T_m_) of polymers.

### Water Contact Angle

Water contact angle (Θ_w_) samples were prepared by solvent casting of polymer from a solution of acetone onto microscope glass slides. Samples were prepared at a concentration of 3 mg/mL, by pipetting 3–4 drops of polymer solution onto the whole surface of the glass slides and letting the solvent evaporate overnight in order to produce a thin film of the polymer. Images were captured using a mobile phone camera through a GoPro Camera App and were analysed using the ImageJ software using a Contact Angle Calculation extension to measure the water contact angles. Video was recorded with the phone camera and still shots were collected using the GoPro app. Samples were measured at a constant temperature (25 °C) with at least three replicates of each measurement recorded at two different time points, t=0 (the exact moment when water touches the polymer surface) and t=5 sec (as soon as the drop settles on the polymer surface).

### Polymer Synthesis

**Scheme 1:**
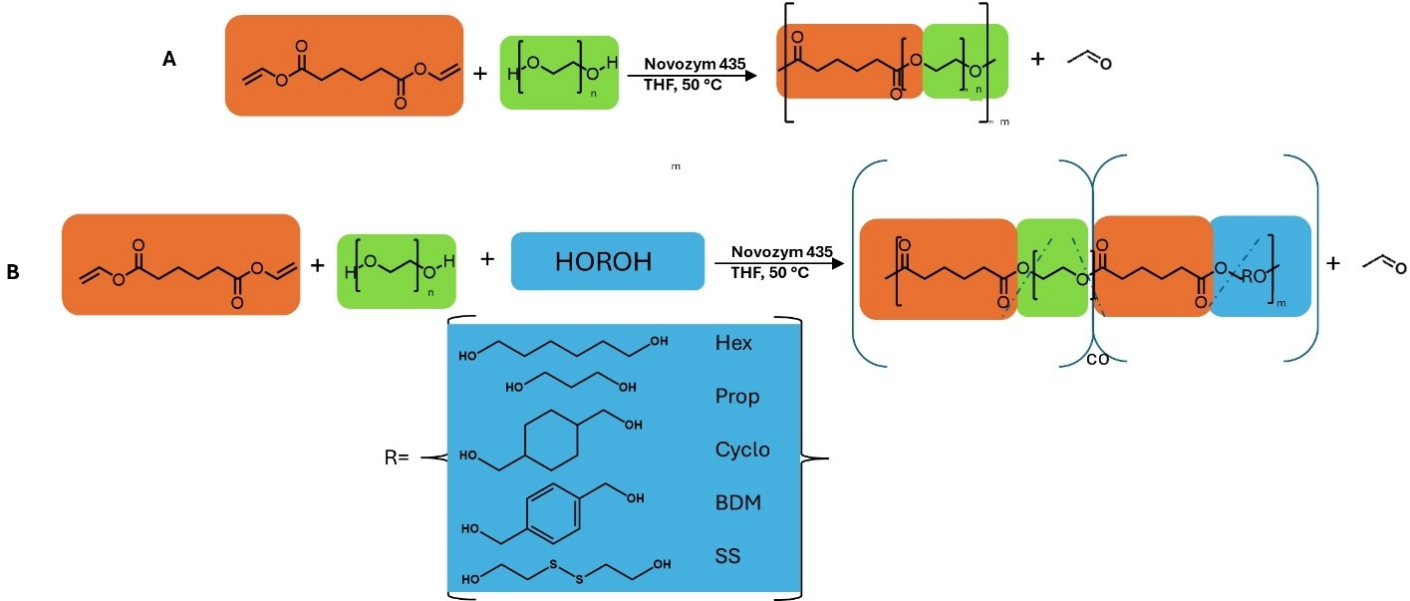
Enzymatic synthesis of A) PEGA and B) PEGA‐X, where R corresponds to the hydrophobic diols. Blue dashed lines indicate that the diols (PEG and hydrophobic diol) in the polyester backbones may not be ideally alternating.

For PEGA synthesis, the previously adopted protocols for PC of polyols with DVA using lipase Novozyme 435 were followed.[^39^, ^40^] In particular, the proposed difference is the replacement of the polyols (glycerol and diglycerol) with PEG chains. The PEG that was used was M_w_=400. In a 20 mL vial, PEG (12.50 mmol) and DVA (12.50 mmol) were weighed and dissolved in THF (10 mL). Following this, Novozyme 435 (0.11 g, 4.4 % w/w with respect to DVA) was added and samples were stirred at 200 rpm and 50 °C for 5 h in a sealed vial pierced with two needles to allow the release of acetaldehyde byproduct. After 5 h, the reaction was terminated by gravity filtration of the enzyme. Excess solvent was then removed under reduced pressure (<200 mbar). The polymer was kept under reduced pressure at 25 °C for a week to remove residual solvent yielding a viscous, pale‐yellow polyester. Polymer conversion was quantitative in all cases, as confirmed by ^1^H NMR spectroscopy, Scheme 1.

According to the previously used protocol,^[39]^ synthesis for hexanediol variants was performed by weighing DVA (12.50 mmol) and the corresponding amounts of PEG and 1,6‐n‐hexanediol into a 20 mL glass vial and dissolving in THF (10 mL) at 50 °C. Two ratios were studied based on previous results.^[39]^ For the 50 : 50 ratio between PEG:Hex (called 50 %), 6.25 mmol of PEG and 6.25 mmol of 1,6‐hexanediol were used to create PEGA‐Hex 50 % (% is hereafter referred to as the amount of 1,6‐hexanediol). Following that, 30 % (PEGA‐Hex 30 %) hexanediol variants were prepared with 3.75 mmol and 8.75 mmol hexanediol and PEG respectively, and the moles of the monomers adjusted according to Moles_PEG_+Moles_Hexanediol_=12.5 mmol.

Following the Hex protocol, an identical procedure was followed for alternative diols, including 1,3‐propanediol, 1,4‐cyclohexanedimethanol, 1,4‐benzenedimethanol, and 2‐hydroxyethyl disulfide. For these diols, only the PEG : Diol 50 : 50 was investigated since it was demonstrated that PEGA‐Hex 50 % had superior properties than PEGA‐Hex 30 % regarding drug encapsulation and NPs stability.

### NPs Formulation and Characterisation

#### NPs Formulation

The ability of PEGA to self‐assemble into nanoparticles (NP) and potentially encapsulate poorly soluble drugs was assessed through the formulation of a solid dispersion, due to the high solubility of PEGA in water. 10 mg of the polymer was dissolved in 1 mL acetone. The solutions were left overnight to evaporate. Then, the samples were resuspended with DI water (4 mL) to a final concentration of 2.5 mg/mL.

#### Dynamic Light Scattering (DLS) Measurements

Particle size and zeta potential were investigated using a Zetasizer Nano ZS spectrometer (Malvern Instruments Ltd., UK). Experiments were performed with a 633 nm laser at a fixed angle of 173°. Samples were equilibrated for 30 s at 25 °C prior to measurement. All samples were measured in triplicate. NPs were prepared at a concentration of 2.5 mg/mL and filtered through 0.22 μm cellulose filter prior to analysis.

#### Coumarin 6and Curcumin Encapsulation Analysis

Dye/polymer solutions were prepared as follows: Dye (coumarin 6 (Coum6) and curcumin were dissolved at 0.5 mg/mL in acetone. Polymer (10 mg) was weighed directly into a glass vial and 1 mL of dye solution was added (polymer : dye ratio of 20 : 1 w/w). Polymer solutions were left overnight to enable acetone evaporation and later resuspended using DI water (4 mL). Nanoparticle‐dye dispersions were filtered through a 0.22 μm filter. Dye controls were filtered and measured in water without the addition of the polymer. Particle size and zeta potential were then determined. Encapsulation of dye was qualitatively determined using fluorescence spectrophotometry, by measuring the fluorescence intensity of the NPs‐dye dispersions at excitation wavelength λ=460 nm and emission wavelength λ=500 nm for coumarin, and at excitation wavelength λ=467 nm and emission wavelength λ=550 nm for curcumin respectively. The apparent water solubility enhancement, of the dyes formulated with our polymers, was semi‐quantitatively evaluated using Equation 1.

$$\Delta F\%=\frac{\Delta F}{F}=\;\frac{\left(F_{NPs}-F_{DYE}\right)}{F_{DYE}}\times 100$$

Equation (1): Measuring the fluorescence of the polymeric systems encapsulating dyes and normalise it using the fluorescence values of the dyes alone, a value for the fluorescence difference can be correlated to the increase of the apparent solubility of the dyes.

F_NPs_=fluorescence signal of NPs formulation with encapsulated dye, normalised by the polymer F.

F_DYE_=fluorescence signal of the free dye in water

### Cytotoxicity Study

#### *In Vitro* Cell Culture

The human mammary adenocarcinoma cell line MCF‐7 was obtained from the American Type Culture Collective (ATCC) and used across a 10‐passage window. Cells were cultured in Dulbecco's Modified Eagle Medium (DMEM) supplemented with 10 % Fetal Bovine Serum (FBS) at 37 °C in a humified incubator with 5 % CO2. Cells were routinely grown in 75 cm^2^ culture flasks to 70 % confluence.

#### *In Vitro* PrestoBlue Metabolic Activity Assay

Cellular metabolic activity was measured using the PrestoBlue viability assay (Thermo Fisher Scientific) as an indication of cytotoxicity. MCF‐7 cells were seeded at 1×10^4^ cells per well in 96 well plates and cultured for 24 h prior to assaying. Cells were exposed to treatments in 100 μl phenol red free DMEM containing 10 % FBS for 24 h. Triton X‐100 was applied at 1 % (v/v) as positive cell death control and medium alone was used as negative control. Following the exposure period, treatments were removed and cells incubated with 100 μl 10 % (v/v) PrestoBlue reagent per well and diluted in phenol red free medium for 60 minutes. The resulting fluorescence was measured on a Tecan Spark 10 M plate reader at an excitation wavelength λ=560 nm and emission wavelength λ=600 nm. Relative metabolic activity is calculated from PrestoBlue data by setting values from the negative control as 100 % and positive control values as 0 % metabolic activity (Equation 2).

$$Relative\;metabolic\;activity=\;\left(\frac{\left(x-Positive\;control\right)}{\left(Negative\;control-Positive\;control\right)}\right)\times 100$$

Equation (2). Calculation of relative metabolic activity. x=treated sample fluorescence value. All values are from fluorescence at 560/600 nm (λex/λem).

#### *In Vitro* LDH Release Test

To study plasma membrane damage in vitro, the extracellular release of lactose dehydrogenase (LDH) enzyme was assessed using the LDH release assay (Sigma Aldrich, TOX7 kit). As above, MCF‐7 cells were seeded at 1×10^4^ cells per well in 96 well plates and cultured for 24 h. Cells were exposed to treatments in 100 μl phenol red free DMEM containing 10 % FBS and received either polymeric formulations, 1 % Triton X‐100 to induce cell lysis or DMEM only to serve as the vehicle control. Following 24 hours exposure, 50 μl of supernatant per well was sampled and transferred to a new 96 well plate for detection of LDH released extracellularly. LDH detection solution was prepared according to the manufacturer's instructions and 100 μl LDH detection reagent added per well to the 50 μL of supernatant sample. The solution was incubated at room temperature protected from light for 25 minutes and the absorbance of the resulting solution measured at 490 nm on a Tecan Spark 10 M plate reader. Relative LDH release was calculated by setting the absorbance signal of 1 % Triton X‐100, assumed to generate full cell lysis, as 100 % LDH release and the background signal generated by LDH detection solution alone as 0 %.

### Statistical Analysis

All experiments were performed in triplicate, and analysis was performed using GraphPad Prism software (v10). Cytotoxicity results for in vitro cell culture were tested for significant differences from the control group (DMEM) using two‐way ANOVA and Dunnett's multiple comparisons post‐hoc test with statistical significance determined at P<0.05.

## Results and Discussion

### PEGA Synthesis

**Figure 1:**
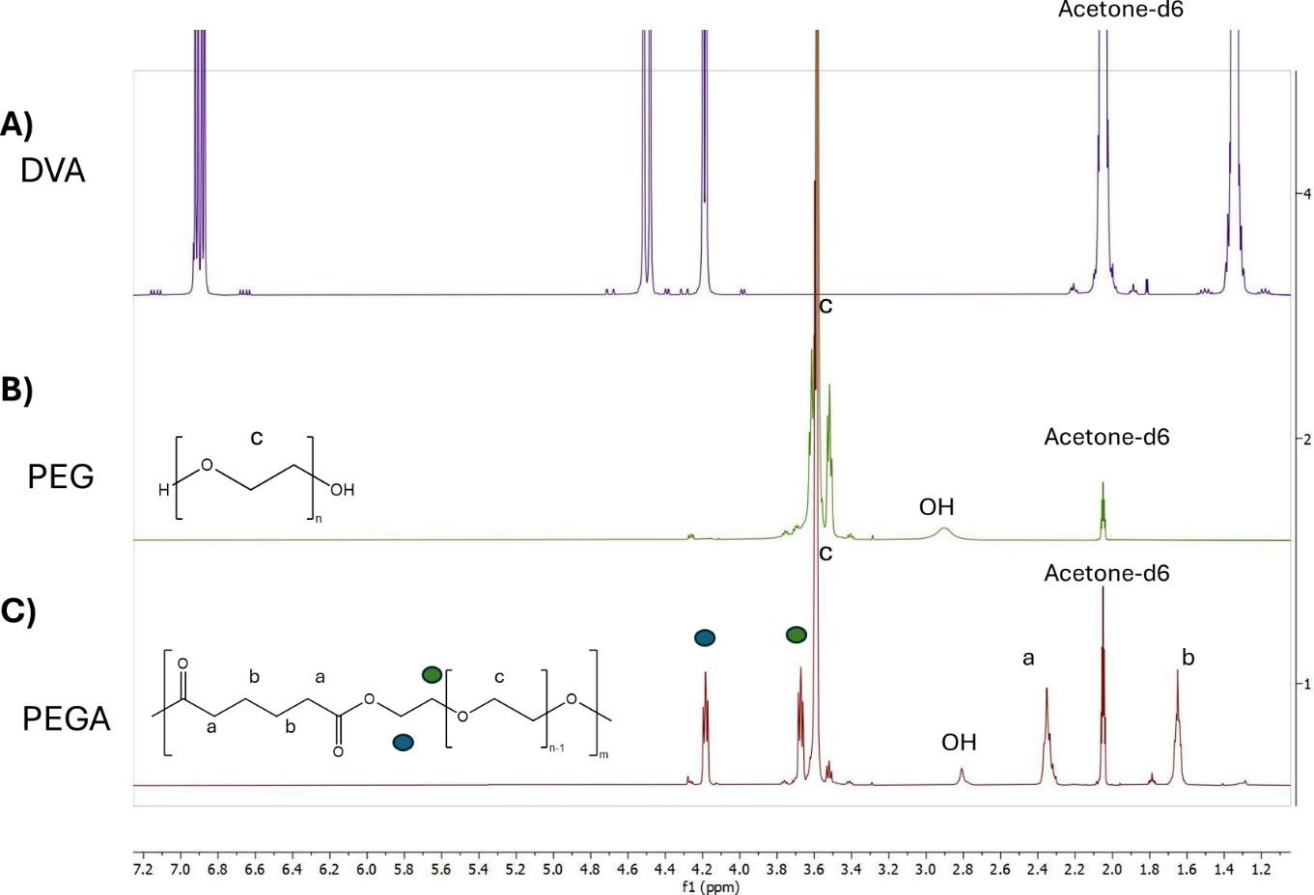
^1^H NMR spectra of A) DVA B) PEG, and C) PEGA. Peaks between 3.6 and 4.3 ppm in spectra C result from the interaction between PEG and DVA that lead to the synthesis of the polyester and are described in depth in Figures S1.

Enzymatic synthesis with an acrylic immobilised lipase resin ‘Novozyme 435’, *Candida antarctica* lipase B (CalB), allows for a controlled polycondensation reaction at mild conditions.[^33^, ^34^] A series of analytical techniques have been used to confirm the success of the polymerisation between PEG400 and DVA to produce the final amphiphilic PEGA polymer. ^1^H‐NMR confirmed the step‐growth polycondensation through the absence of characteristic vinyl peaks of DVA initially present in the DVA monomer at 4.59, 4.87, and 7.29 ppm (Figure 1)^[44]^ and the presence of new peaks at 3.60 and 4.20 ppm related to methylene protons of the −OC*H_2_*−C*H_2_* PEG unit directly linked to the ester bond. The nature of these peaks has been also confirmed by HSQC and HMBC 2D‐NMR analysis to further corroborate the formation of an ester bond during the polymerisation process (Figure 1 and Figure S1). In addition, the incorporation of adipate was confirmed by the protons of the −C*H_2_* peak at 2.35 ppm adjacent to carbonyl and the alkyl −C*H_2_* peak at 1.65 ppm (Figure 1). GPC analyses further proved the polymerisation reaction as the average molar masses increased from M_n_ 3.3×10^2^ g/mol and *Ð*=1.25 for free PEG to M_n_ of 4.0×10^3^ g/mol and *Ð*=1.52 (Table 1). The broader molar mass distribution, expected for a step growth polymerisation, also demonstrated polymerisation had occurred. Moreover, the produced PEG‐polyester displayed thermal properties different to PEG alone: T_m_ dropped from 5.20 °C for PEG to approximately −6 °C for the polymer, whilst PEGA displayed a T_g_ value of approximately −58 °C (Table 1 and DSC thermograms section in SI), whilst PEG alone does not show a glass transition within the explored temperature range.

**Table 1:** Number average molar mass (M_n_), *Ð*, T_g_, T_m_, and ΔH_m_.

Polymer	M_n_ (gmol^−1^)^[a]^	D^[a]^	T_m_ (°C)^[b]^	ΔH_m_ (Jg^−1^)^[b]^	T_g_ (°C)^[b]^
	THF				
PEG400	3.3×10^2^	1.25	5.20	107.2	N/A
PEGA	4.0×10^3^	1.52	−5.99	25.45	−57.37
PEGA‐Hex 30 %	3.6×10^3^	1.93	−8.61	39.62	−60.35
PEGA‐Hex 50 %	3.5×10^3^	1.90	−9.11	26.23	−61.37
PEGA‐Prop 50 %	1.6×10^3^	2.47	N/A	N/A^*^	−59.88
PEGA‐Cyclo 50 %	2.0×10^3^	2.23	N/A	N/A^**^	−54.98
PEGA‐BDM 50 %	2.4×10^3^	1.60	N/A	N/A	−54.41
PEGA‐SS 50 %	2.9×10^3^	1.94	N/A	N/A	−54.43

^*^a weak endothermic transition can be observed at around 0 °C, but difficult to assigned as a pure melting transition (DSC thermograms section in SI). ^**^a weak exothermic peak could be observed, at around 0 °C, but no subsequent melting peak could be observed (DSC thermograms section in SI).

### Synthesis of Hexanediol Variants

**Figure 2:**
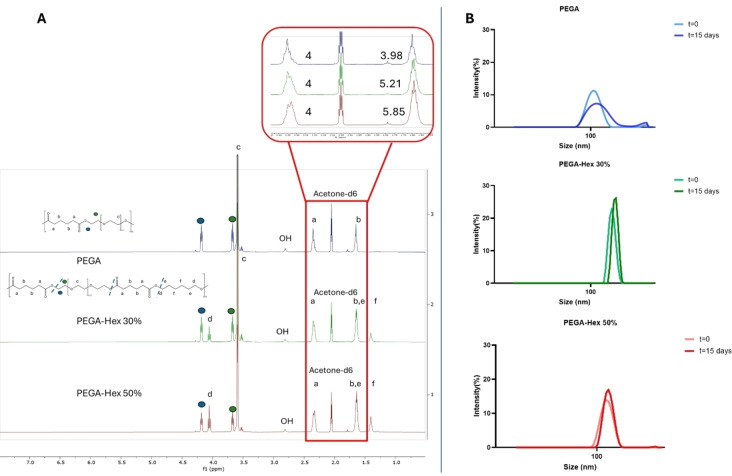
Screening for the optimal ratio of PEGA:Hex for the hexanediol variants. A. ^1^H NMR spectra shows successful synthesis of the hexanediol variants. The inlay (red) shows integration of adipic peaks at 2.36 ppm compared to the overlapping peaks at 1.65 ppm. For simplicity, the integration for the adipic peak was set at 4. B. DLS graphs for an initial stability evaluation of NPs up to t=15 days illustrating that PEGA‐Hex 50 % creates the smallest and more stable NPs.

The stoichiometric addition of 1,6‐hexanediol as a functionalised diol was adopted to alter the amphiphilic balance as well as to investigate the possible range of chemical and physical properties of the final PEGA derivatives. Following the previous ^1^H NMR observations for PEGA, it is also possible to observe the appearance of new peaks traceable to the protons of the PEG unit linked to the adipic moiety, as well as absence of characteristic vinyl protons and broadening of the adipic methylene protons (Figure 2A and Figure S2). Furthermore, the incorporation of hexane‐1,6‐diol is confirmed by additional peaks at 1.40 (alkyl −C*H_2_*) and 4.05 (ester adjacent −C*H_2_*) ppm, and the overlapping peaks at 1.65 ppm (Figure 2A and Figure S2). The peak at 1.65 ppm is composed of 4 adipic methylene protons and 4 methylene protons of hexanediol. The integrals of these protons are consistent with the molar ratios of diols used in the polymer syntheses (Figure S2). When the adipic peak at 2.36 ppm is set equal to 4 protons, for PEGA‐Hex 50 % the expected integral is 6, which is the result of 4 (adipic) protons and 4×0.5 equivalents −C*H_2_*. Following that, for PEGA‐Hex 30 %, the expected integral at 1.65 ppm is 5.2, which is the result of 4 (adipic) protons and 4×0.3 equivalents −C*H_2_* of hexanediol proton (Figure 2A). For PEGA‐Hex 50 %, the measured integrals of 5.87, which agrees with the expected value. In addition, the same applies to PEGA‐Hex 30 %, for which the measured value is 5.21.

Following the ^1^H NMR, the successful synthesis of the hexanediol variants was also confirmed by GPC analysis. Whilst GPC chromatograms showed a broad (1.5≤*Ð*≤2.10) polymeric peak for each derivative, hexanediol variants displayed a broader peak than PEGA (Table 1), due to the presence of oligomers visible in the chromatogram (Figure S3). However, the presence of oligomers, along with the broad dispersity, is to be expected for step‐growth polymerisations.[^39^, ^40^, ^45^] Although molar masses remained approximately the same with incorporating the hydrophobic segment, a clear trend can be observed: Increasing the amount of hexanediol resulted in a slight decrease in the molar mass. This is due to higher solubility of PEG in THF (GPC mobile phase) compared to hexanediol, driving up the molecular mass of PEGA compared to the hexanediol variants. Moreover, when increasing the Hex amount above 30 %, although polymerisation is still complete, molar mass further decreases, suggesting the production of more oligomers with reduced solubility in THF. Both variants displayed lower T_g_, T_m,_ and ΔH_m_ values when compared to the PEGA polymer (Table 1 and DSC thermograms section in SI). This is due to the reduction of PEG molar ratio, as well as the introduction of a new, more crystalline Hex disrupting PEG's recrystallisation propensity. Although the ratio of the hydrophobic segment does not significantly affect the polymers’ molar mass, it influences the NPs formation by nanoprecipitation and related stability over time (up to 15 days). Even though the NPs made by PEGA have smaller sizes than the hexanediol variants (Figure 2B), the stability of the NPs over time is enhanced by the incorporation of the hydrophobic diol, especially at the ratio of PEGA : Diol 50 : 50 (Figure 2B). For this reason, it was decided to investigate the incorporation of a series of hydrophobic diols, at the optimal ratio of 50 : 50.

### Synthesis of Diol‐Modified PEGA Variants

**Figure 3:**
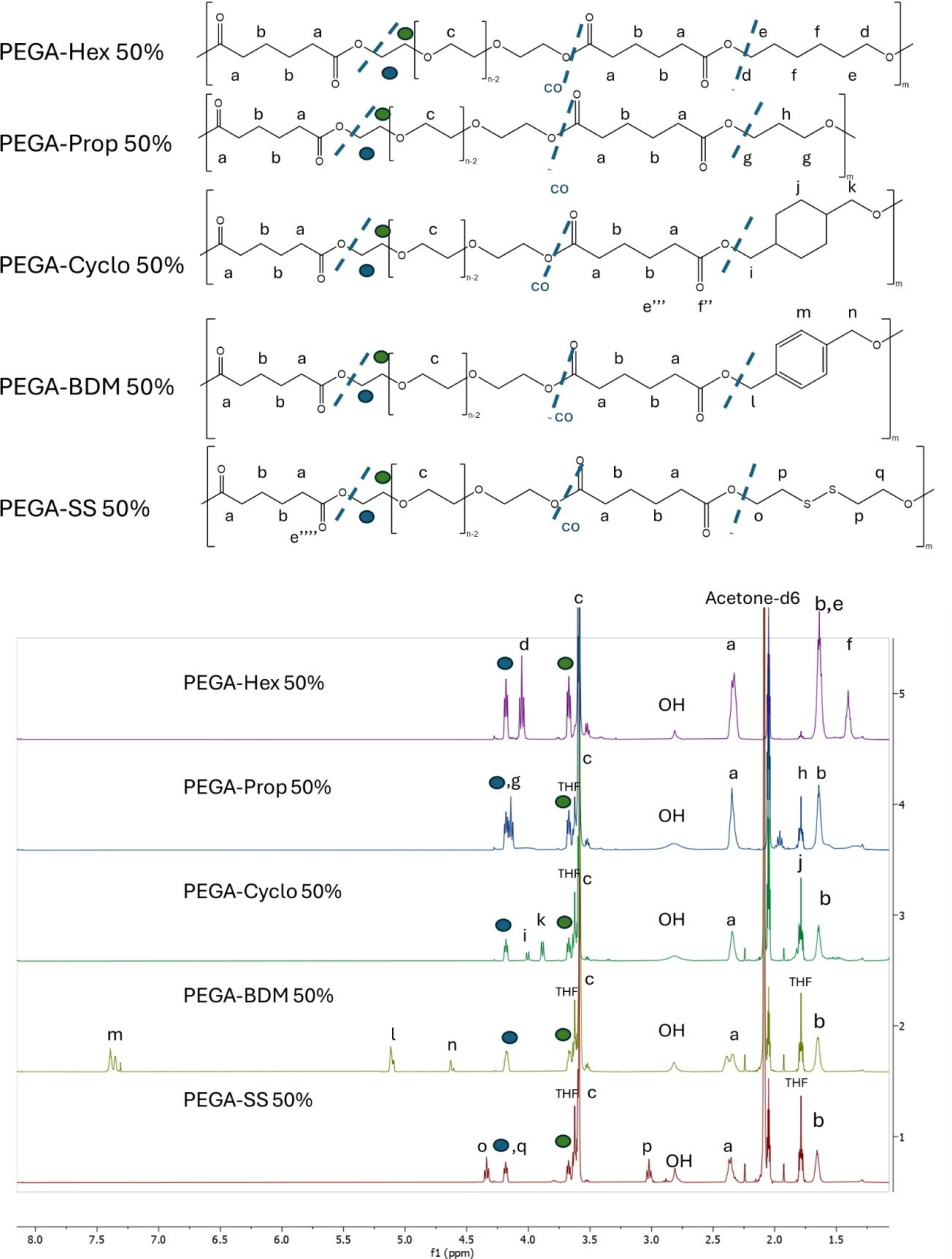
^1^H NMR spectra for PEGA modifications. Inset shows the measured integrals for the peak at 1.65 ppm which indicates the molar ratios of diols used in the polymer syntheses. Blue dashes represent that the diols in the polyester backbones may not be repeating sequentially.

The insertion of a range of hydrophobic moieties into the backbone aimed at modifying the amphiphilic balance of the polymeric backbone and incorporating additional functionalities. All ^1^H‐NMR results (Figure 3) for all the PEGylated polymers are in agreement with both experimental results on PEGA and PEGA‐Hex as well as previous results in the literature, where enzymatic PC was employed to synthesise functionalisable and biodegradable polyesters.[^39^, ^45^] The length of such polyester's hydrophobic chain can have a significant effect on its properties, including hydrophobicity, foaming ability, and mechanical strength.[^46^, ^47^] In particular, a shorter chain length diol, 1,3‐propanediol, replaced 1,6‐hexanediol to demonstrate whether the chain length will influence such properties, as well as encapsulation ability. In addition, cyclic moieties can also induce a profound effect on polymer properties. Cyclic compounds can enhance the biological activity,^[48]^ whilst aromatic rings can influence the cell uptake of compounds.^[49]^ Therefore, the incorporation of 1,4‐cyclohexanedimethanol and 1,4‐benzenedimethanol in the polyester backbone was investigated for the influence of the NPs formation and drug encapsulation. Finally, NPs formation, stability and encapsulation ability of PEGA bearing 2‐hydroxyethyl disulfide, a readily available and well‐known redox‐responsive linear diol, were tested.^[45]^

All the variants showed the presence of the same new peaks reported earlier, as well as the characteristic peaks enlargement and shifting observed for PEGA. The incorporation of 1,3‐propanediol is confirmed by the additional peaks at 4.15 (ester adjacent −C*H_2_*) overlapping with the PEG ester adjacent methylene protons, as there was an increase in the integral and at 1.60 (alkyl −C*H_2_*) (Figure 3). The incorporation of 1,4‐cyclohexanedimethanol is confirmed by the additional peaks at 4.20 (ester adjacent −C*H_2_* protons), 3.9 ppm (ester adjacent−C*H_2_* protons), and 1.64 ppm (cyclohexyl protons) (Figure 3). The incorporation of 1,4‐benzenedimethanol is confirmed by the additional peaks at 7.4 (aromatic protons), 5.12 (ester adjacent −C*H_2_* protons), and 4.20 ppm (ester adjacent −C*H_2_* protons) (Figure 3). The incorporation of 2‐hydroxyethyl disulphide is confirmed by the additional peaks at 4.30 (ester adjacent −C*H_2_* protons), 4.18 (ester adjacent −C*H_2_* protons), and 3.0 (sulfide adjacent −C*H_2_* protons) (Figure 3).

For all the diol‐modified PEGA derivatives, GPC traces showed broad (1.5≤*Ð*≤2.5) (Figure S3) polymeric peaks with ranging molar masses, from 1600 up to 4000 g mol^−1^ (Table 1) and traces of oligomers, as previously observed in literature for similar enzymatically synthesised polymers 40,41,47, Figure S1. PEGA‐Prop 50 % demonstrates the lowest *M_n_* value, as expected due to the shorter chain of propanediol compared to the other diols. Polyesters containing hexanediol moieties yield the highest *M_n_* values. Again, this may be due to higher solubility of hexanediol in THF in comparison to other hydrophobic diols. Adding to that trend, diols with aromatic and non‐aromatic rings provide *M_n_* values higher than the propanediol variants but lower than the hexanediol and the disulfide variants, aligning with similar polymers from literature.^[45]^ Interestingly, all the variants, apart from Hex, completely prevent the recrystallisation of PEG, generating amorphous polymers with T_g_ values ranging from approx. −60 °C to approx. −54 °C (DSC thermograms section in SI). Such values demonstrate high chain flexibility at room temperature, which can be exploited in the formation of NPs with higher penetration properties in biological environments.^[50]^

**Figure 4:**
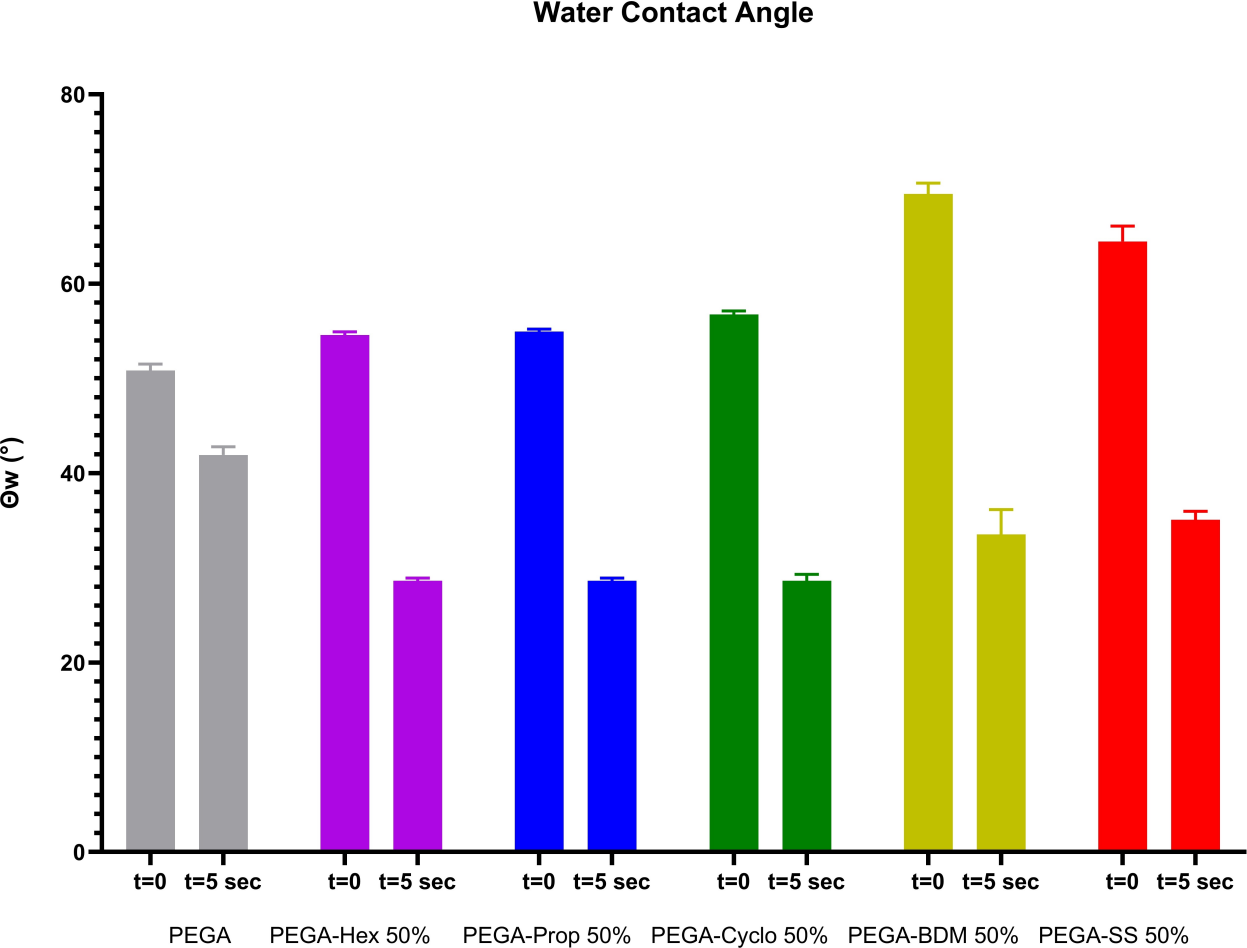
Results of the Water contact angle measurements. Measurements were taken at two different time points; t=0 s (as soon as the water droplet touched the polymer surface) and t=5 s (when the water droplet settled on the polymer surface).

To assess the effect on surface hydrophobicity of synthesised polyesters, and consequent PEG content reduction, water contact angle (Θ_w_) measurements were performed. It has been observed that the Θ_w_ measured immediately after the water drop came in contact (t=0) with the polymeric surfaces of the PEGA variants were higher than the angle for PEGA alone (50.83°) (Figure 4). This agrees with the reduction of PEG content and introduction of more hydrophobic moieties with the aromatic BDM recording the highest value of circa 69.5° (Figure 4). However, it can be noticed that all the Θ_w_ measured are well below 90°, which is considered the conventional threshold for defining a surface as hydrophobic.^[51]^ Such results hint at amphiphilicity of the polyesters, which was then confirmed by a considerable dropping of Θ_w_ after 5 seconds of contact between the water drop and the surface. This hydrophilicity enhancement is attributed to the spontaneous rearrangement of the hydrophilic PEG chains towards water. This is particularly evidenced by high drops (up to −30° from the initial value) in Θ_w_ being observed for all variants. On the other hand, PEGA wettability did not change with time, due to higher PEG ratio and less intramolecular rearrangement towards the water drop.

### NPs Formation and Stability

NPs size is one of the most crucial properties influencing cellular uptake and absorption of APIs across the gastrointestinal (GI) tract.^[52]^ Generally, the optimal size of NPs for drug delivery depends on various attributes such as the type of drug, the route of administration, and the target organ or tissue.[^53^, ^54^] In this regard, it has been reported that NPs with sizes below 300 nm can result in enhanced absorption in the systemic circulation.[^55^, ^56^] Ideally, the size range of nanoparticles for drug delivery is between 10 and 200 nm.^[57]^

All synthesised polymers have been tested for their ability to form NPs according to protocol previously described in literature.^[4]^ All NPs demonstrate comparable sizes around or below 200 nm, ranging from approximately 146 nm to 210 nm with low size distribution (PDI) values (Table 2).

**Table 2:** Size and PDI values for the diol‐modified pegylated NPs.

Polymer	Average size/nm	PDI
PEGA‐Hex 50 %	171.4±1.8	0.21±0.03
PEGA‐Prop 50 %	145.7±0.4	0.04±0.03
PEGA‐Cyclo 50 %	183.9±3.9	0.13±0.02
PEGA‐BDM 50 %	209.8±2.8	0.06±0.02
PEGA‐SS 50 %	170.2±0.6	0.02±0.00

While PEGA‐Prop 50 %, synthesised using the shortest diol, created the smallest NPs, PEGA‐Cyclo 50 % and PEGA‐BDM 50 % which bear a bulkier aliphatic and aromatic ring respectively, formed larger NPs than the other polymers (Table 2), as expected. Although different diol variants gave comparable NP sizes, PDI varied, with PEGA‐Prop 50 %, PEGA‐BDM 50 %, and PEGA‐SS 50 % having the narrowest size distributions (Table 2).

**Figure 5:**
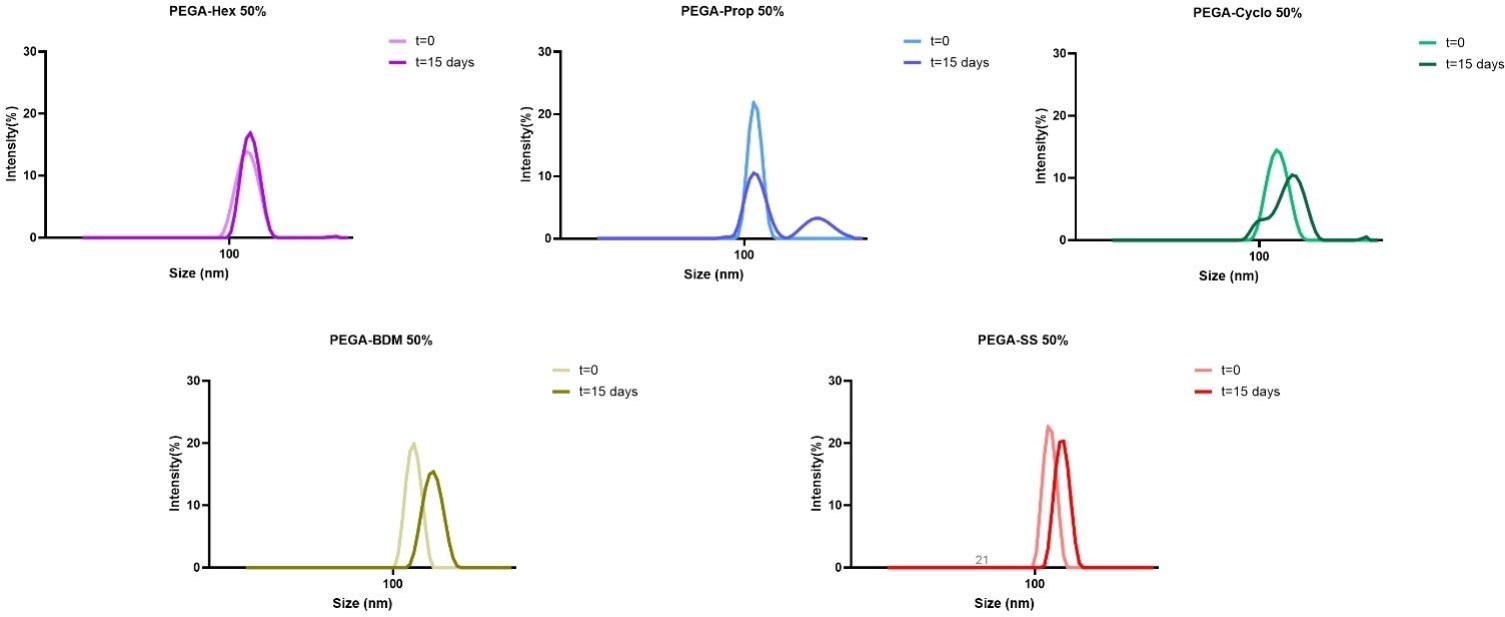
DLS sizes trend showing NPs stability over time up to 15 days for all the PEGA‐variants.

The stability of NPs is noticeably affected by the diol‐modification, Figure 5. PEGA‐prop has a shorter hydrophobic chain and thus it formed a looser core of NPs and had lower stability than other polymers. While PEGA‐BDM NPs would be more stable compared to PEGA‐Cyclo since aromatic ring may form π‐π stacking in the core of NPs.^[58]^ For PEGA‐Hex and PEGA‐SS comparable features could be envisaged as they have similar aliphatic chain length. This was confirmed by similar NPs sizes (Table 2) and overall, a good stability, for both systems, after 15 days (Table S1).

### Model Drug Encapsulation Screening

To investigate the use of these polymeric systems as drug delivery carriers, an initial encapsulation study was conducted using two hydrophobic compounds as model drugs; Coumarin 6 (Coum6),^[59]^ following previous protocols[^39^, ^40^] and Curcumin^[60]^ by exploiting fluorescence properties of both.[^61^, ^62^] Both dyes possess drug characteristics, that render them ideal systems for this qualitative encapsulation study with LogP values of 4.9^[40]^ and 3.2^[63]^ respectively. Such molecules can mimic the behaviour of lipophilic APIs due to as their water‐insolubility and biological properties, including anticancer and antimicrobial activities.[^64^, ^65^, ^66^]

**Figure 6:**
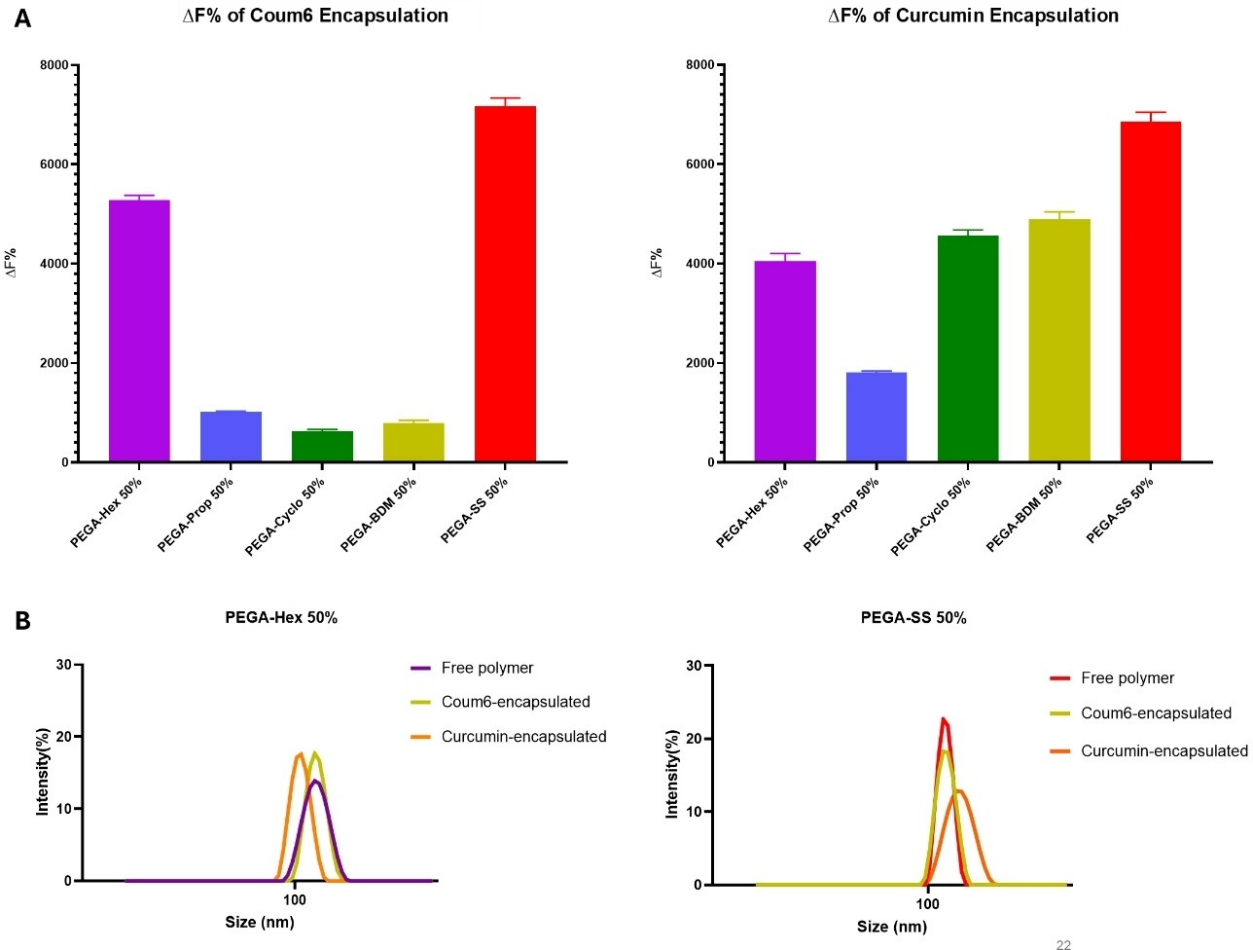
ΔF% ranking of the Coum6 and Curcumin encapsulated systems of PEGA‐Hex 50 %, PEGA‐Prop 50 %, PEGA‐Cyclo 50 %, PEGA‐BDM 50 %, and PEGA‐SS 50 %. B. DLS traces showing the size distribution of the PEGA‐Hex 50 % and PEGA‐SS 50 % as free polymers against the corresponding polymers after encapsulation of Coum6 and Curcumin.

This study aims to analyse the apparent water solubility enhancement of the two model hydrophobic drugs when nano‐formulated with the synthesised PEGylated systems. In this way, insight can be provided into the selection of the best variants to be used as drug delivery vehicles. ΔF% could be directly related to the enhancement of the water apparent solubility of the hydrophobic dyes.^[39]^ Based on Equation (1) the synthesised polymers and the controls are ranked quickly and in a semi‐quantitative way according to their ΔF% value (Figure 6A).

As expected from different LogP values, it was observed that the two drug models had different apparent water solubilities. Among all polymers, PEGA‐SS had the highest entrapment efficiency of both dyes, due to a balance between chain flexibility and hydrophobicity, which was also observed for similar polymers based on glycerol with other hydrophobic model dyes.^[67]^ Coum6 is more hydrophobic (higher LogP) and sterically bulkier than curcumin, which can explain differences in entrapment ability. Demonstratively, a higher amount of curcumin, could be entrapped into PEGA‐Prop, PEGA‐Cyclo, and PEGA‐BDM NPs compared to Coum6 (Figure 6A). Despite this, Coum6 to interacted more with PEGA‐Hex than curcumin, due to a higher compatibility between hydrophobicity of both Coum6 and Hex. PEGA‐Prop had minimal efficiency on the solubilisation of drugs, especially curcumin, due to a more hydrophilic NP core caused by the shorter aliphatic chain. Sterically hindered cyclohexane (PEGA‐Cyclo) and benzene containing polyesters (PEGA‐BDM) showed limited but comparable solubilisation capacity of both models. Overall, PEGA‐Hex and PEGA‐SS can enhance the water apparent solubility of both Coum6 and curcumin. Finally, only slight size variations could be observed for the best performing NPs systems after drug encapsulation. No size variations were observed for either of the two polymeric NPs in presence of Coum6, while minimal shrinkage, for PEGA‐Hex, and minimal enlargement, for PEGA‐SS in presence of curcumin could be observed (Figure 6B).

### NPs *In Vitro* Toxicity Testing

**Figure 7:**
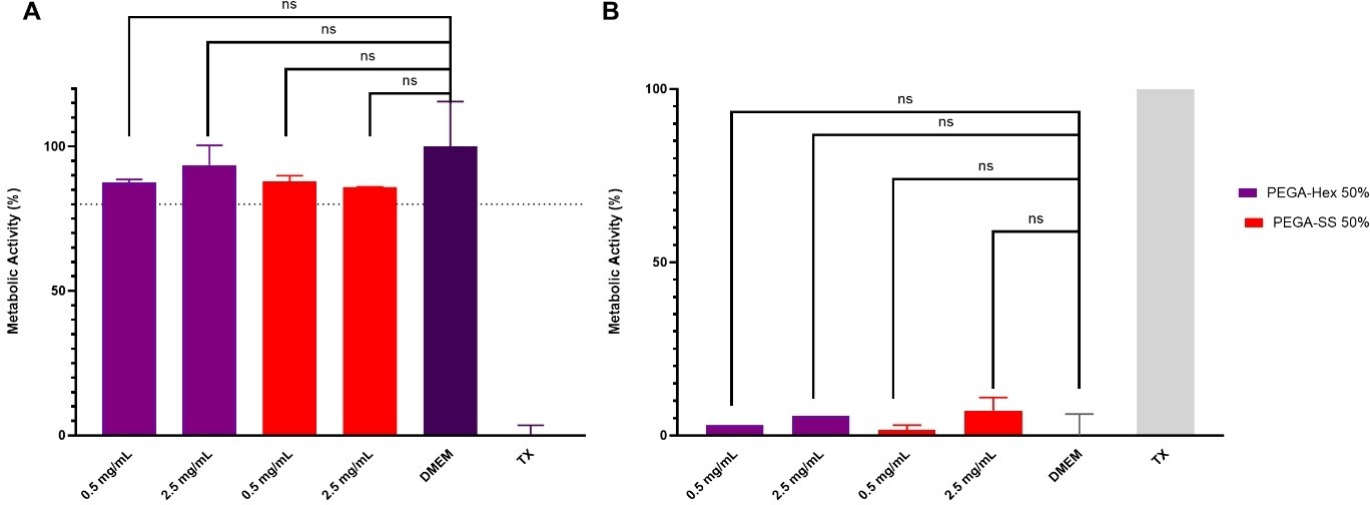
Cytotoxicity of tested polymeric systems in MCF‐7 breast cells assessed via **A**. metabolic activity using PrestoBlue assay and **B**. LDH release assay. Cells were exposed for 24 hours to varying concentrations of polymeric systems and DMEM culture media as vehicle control and 1 % (v/v) Triton X‐100 (TX) as cell death‐inducing control. Data presented as mean ± S.D. Statistical analysis using a two‐way ANOVA test determined no significant difference between test systems and DMEM control in both assays (P >0.05).

To assess the cytocompatibility of the best‐performing NPs systems, PEGA‐Hex 50 % and PEGA‐SS 50 %, human breast MCF‐7 cells were exposed for 24 hours to two concentrations of the polymeric systems: 0.5 mg/mL and 2.5 mg/mL (Figure 7). The resulting metabolic activity was then probed to indicate cell viability (Figure 7A) and the extracellular release of LDH enzyme was measured to evaluate potential plasma membrane damage (Figure 7B). The data demonstrate that the applied systems are non‐toxic to cells at both tested concentrations (≤2.5 mg/mL), as indicated by no statistical or substantial change in cellular metabolic activity or LDH release when compared to the vehicle control (DMEM).

## Conclusions

This work investigated the potential use of a one‐pot, one‐step enzymatic polycondensation process to synthesise a small library of amphiphilic PEG‐adipate variants with enhanced physiochemical and nanoproduction properties compared to free PEG. This was achieved by varying the nature of the hydrophobic and functionalised diols synthesised together with PEG. In particular, the incorporation of such diols was examined for their impact on nanoparticle properties, such as formation, stability, and drug encapsulation. One hydrophobic variant, Hex, served as a model to screen the effect of PEG:functionalised diol feed‐ratio upon self‐assembly ability, to select the best performing molar ratio. It was clearly shown that the stability of NPs is significantly influenced by the nature of the diols. Specifically, PEGA‐prop, with its shorter hydrophobic chain, formed a looser NPs core, resulting in lower stability compared to other polymers. In contrast, PEGA‐BDM NPs demonstrated greater stability than PEGA‐Cyclo NPs, likely due to the aromatic ring's ability to form π‐π stacking interactions within the NP core. PEGA‐Hex and PEGA‐SS exhibited comparable features, attributed to their similar aliphatic chain lengths, which was confirmed by the similar NPs sizes and overall good stability that was observed for both systems for a timeframe of up to 15 days. Self‐assembly and the encapsulation of water‐insoluble model drugs were proven to be directly related to the nature of the added functionalised diol. Combined with the absence of cytotoxicity in vitro experiments, this work shows promising potential for further screening of these PEGylated polymers as nano‐drug delivery carriers. As a preliminary result, PEGA‐Hex 50 % and PEGA‐SS 50 % proved to be the best candidates, by balancing enhanced NP stability and desirable ‐dye encapsulation. However, a slightly increase in sizes, with time, was observed also for these two best‐performing polymers.

The initial water contact angle (Θw) measurements for the PEGA variants were higher than that of PEGA alone (50.83°), with the aromatic BDM variant recording the highest value at approximately 69.5°. Despite this, all Θw values were below 90°, indicating that the surfaces were not hydrophobic. These results suggest the amphiphilic nature of the polyesters, which was confirmed by a significant decrease in Θw after 5 seconds of contact with water. This decrease, up to 30° from the initial value, is attributed to the spontaneous rearrangement of hydrophilic PEG chains towards the water. In contrast, PEGA's wettability remained unchanged over time due to its higher PEG content and reduced intramolecular rearrangement. While the high hydrophilicity led to lower nanoparticle (NP) stability in this study, with only two variants showing stability for up to two weeks, this property, combined with the water solubility and thermal properties of the materials, can be exploited for the production of amorphous solid dispersions. The enzymatic synthesis of these polymers further enhances their sustainability and biodegradability. By limiting the PEG content, this powerful technology can be advanced towards a more sustainable future. Additionally, the possibility of introducing hybrid systems with a limited fraction of PEG, utilising other polyols like glycerol, opens new avenues for creating innovative and environmentally friendly drug delivery systems. The promising results reported in this work highlight the potential for further exploration into the design and modification of biodegradable PEGylated polyesters, paving the way for more effective and sustainable drug delivery nano‐systems with diverse responsiveness.

## Conflict of Interests

The authors declare no conflict of interest.

1

## Supporting information

As a service to our authors and readers, this journal provides supporting information supplied by the authors. Such materials are peer reviewed and may be re‐organized for online delivery, but are not copy‐edited or typeset. Technical support issues arising from supporting information (other than missing files) should be addressed to the authors.

Supporting Information

## Data Availability

Data is available on request from the corresponding authors.
