# Supplementary material for: Enzymatic Synthesis of Functional PEGylated Adipate Copolymers
Source: Chempluschem. 2025 Mar 12;90(5):e202400668. doi: 10.1002/cplu.202400668 (PMC12105458; doi:10.1002/cplu.202400668)
Supplement: Supplementary file 1 — Supporting Information [file CPLU-90-e202400668-s001.pdf]

ChemPlusChem

Supporting Information

Enzymatic Synthesis of Functional PEGylated Adipate Copolymers

Eleni Axioti, Emily G. Dixon, Thomas Jepras, Fen Tin He, Peter J. V. Hartman, Bradley Hopkins, Vincenzo Di Bari, Jiraphong Suksiriworapong, Valentina Cuzzucoli Crucitti, Luciano Galantini, Iolanda Francolini, Robert J Cavanagh, and Vincenzo Taresco*

Support Information

Enzymatic Synthesis of Functional PEGylated Adipate Copolymers

Eleni Axioti,[a] Emily G. Dixon,[a] Thomas Jepras,[a] Fen Tin He,[a] Peter James Vitezslav Hartman[b] , Bradley Hopkins,[a] Vincenzo Di Bari,[b] Jiraphong Suksiriworapong,[c] Valentina Cuzzucoli Crucitti,[d] Luciano Galantini,[e] Iolanda Francolini,[e] Robert J. Cavanagh,[f] and Vincenzo Taresco*[a]

[a] School of Chemistry, University Park, Nottingham NG7 2RD, United Kingdom

[b] Division of Food, Nutrition and Dietetics, School of Biosciences, University of Nottingham, Sutton Bonington Campus, LE12 5RD, UK

[c] Department of Pharmacy, Faculty of Pharmacy, Mahidol University, Bangkok, 10400, Thailand

[d] Centre for Additive Manufacturing, Department of Chemical and Environmental Engineering, University of Nottingham, University Park, Nottingham NG7 2RD, United Kingdom

[e] Dept. of Chemistry, Sapienza University of Rome, Piazzale A. Moro 5, Rome 00185, Italy

[f] School of Pharmacy, University Park, Nottingham NG7 2RD, United Kingdom

*corresponding author, email: vincenzo.taresco@nottingham.ac.uk

A)

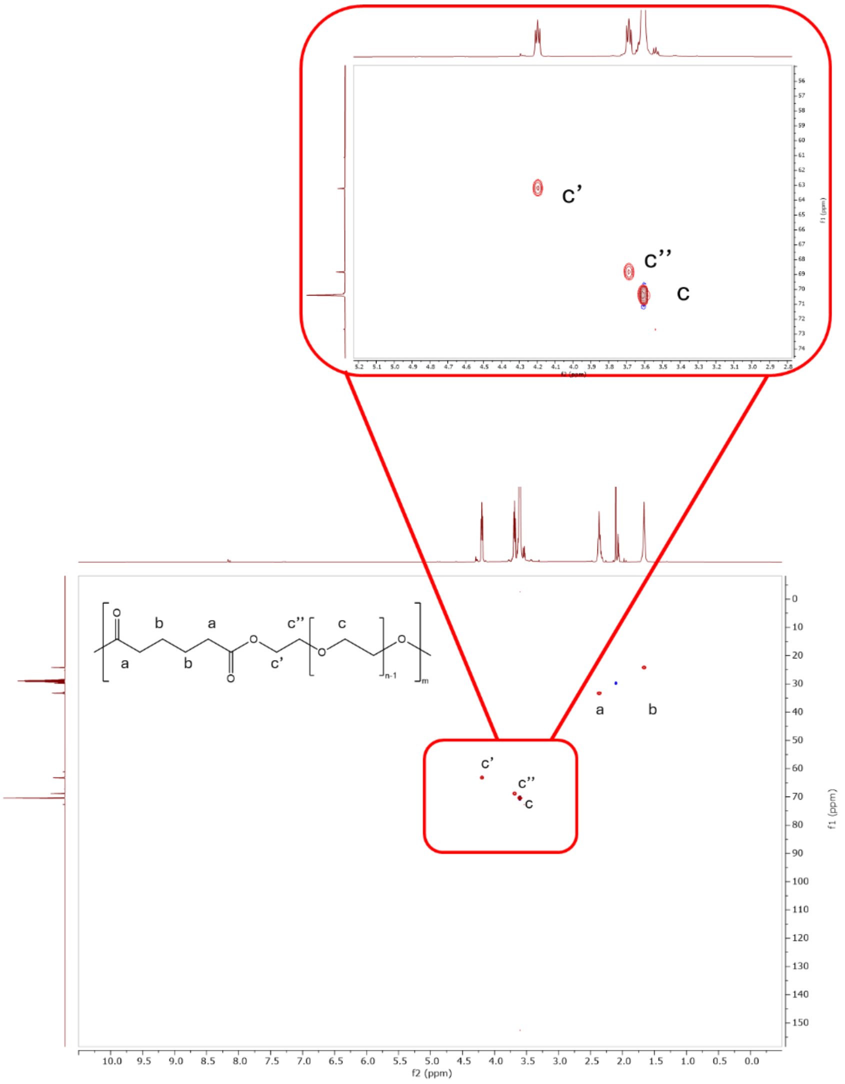

B)

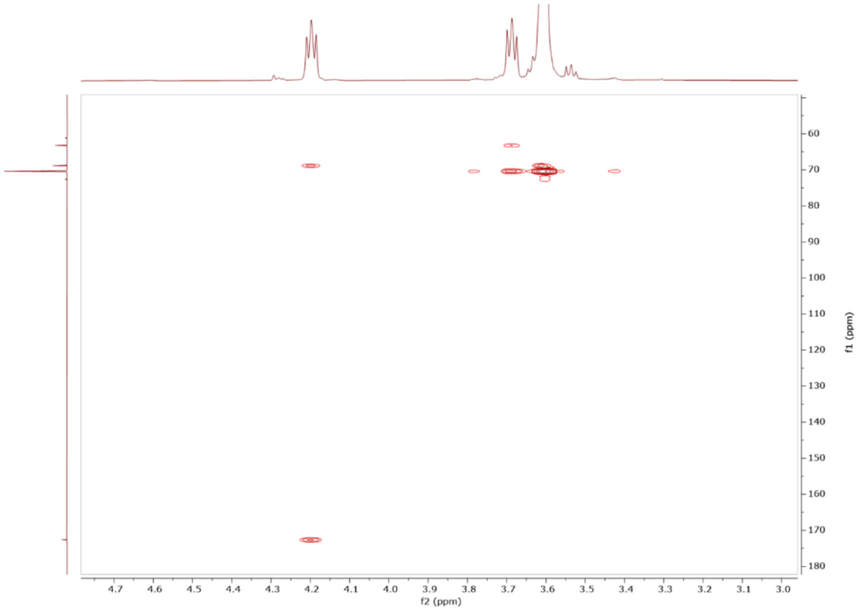

Figure S1: A. ^{1}H-^{13}C HSQC NMR spectrum of PEGA and B) ^{1}H-^{13}C HMBC NMR spectrum of PEGA, highlighting the peaks related to the PEG units connected to the ester group.

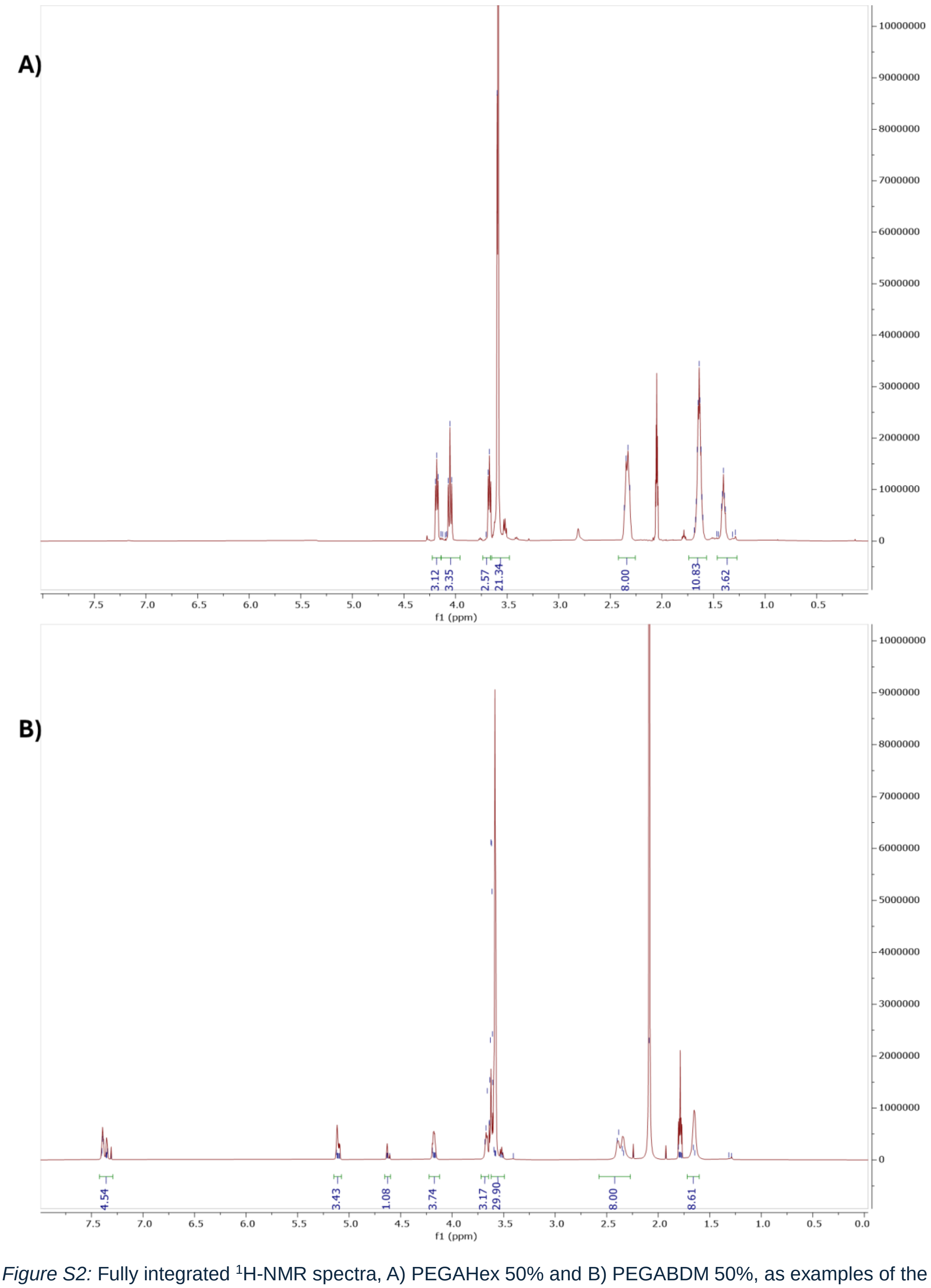

Figure S2: Fully integrated ^{1}H-NMR spectra, A) PEGAHex 50% and B) PEGABDM 50%, as examples of the different variants.

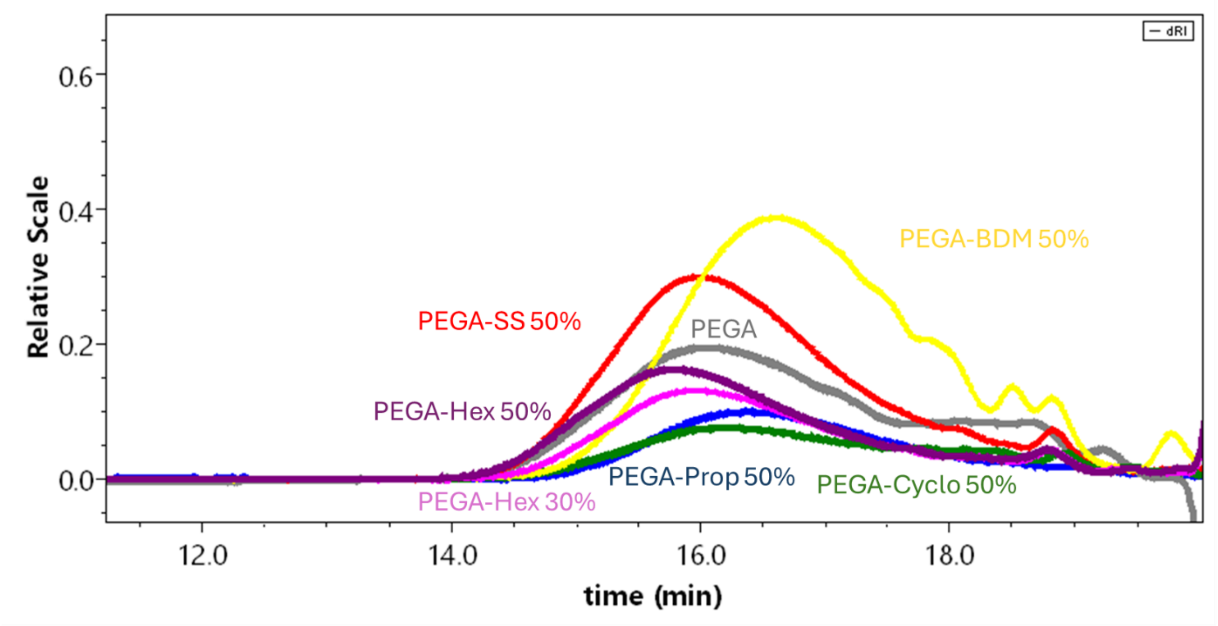

Figure S3: GPC traces for all the synthesised pegylated polymers.

DSC thermograms

DSC thermograms (heating cycle) of polymer variants.

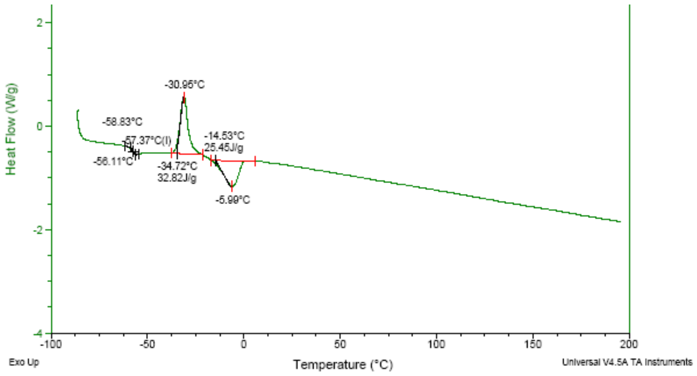

PEGA

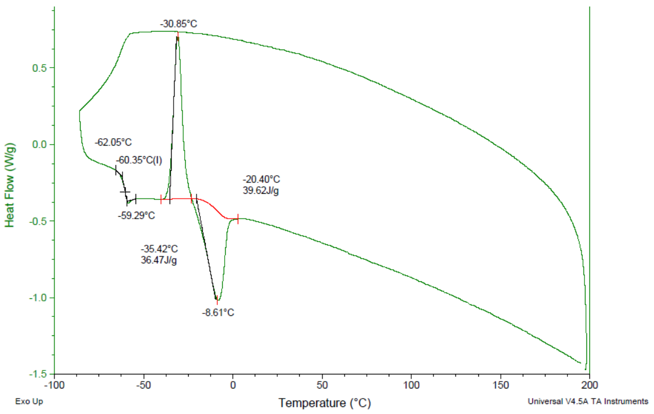

PEGA-Hex 30%

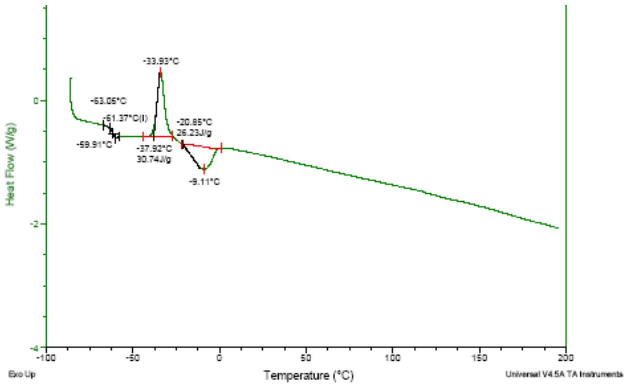

PEGA-Hex 50%

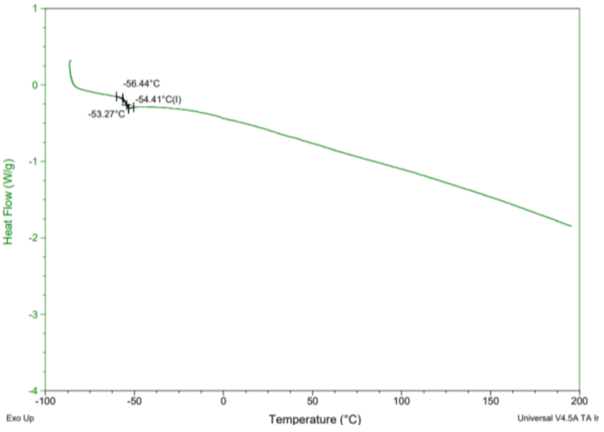

PEGA-BDM

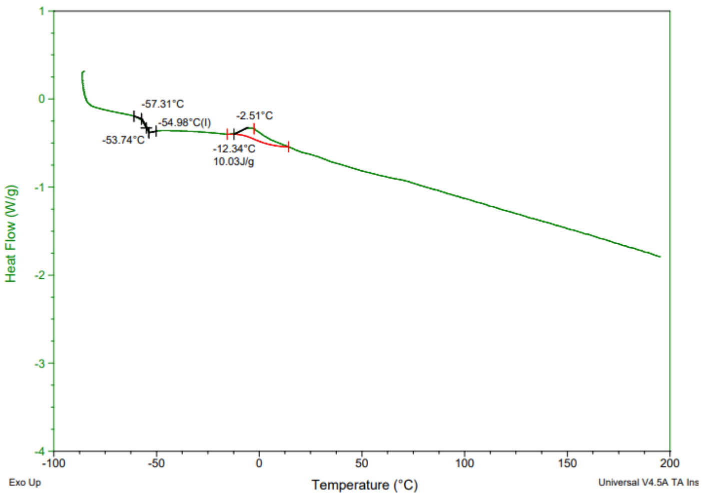

PEGA-Cyclo

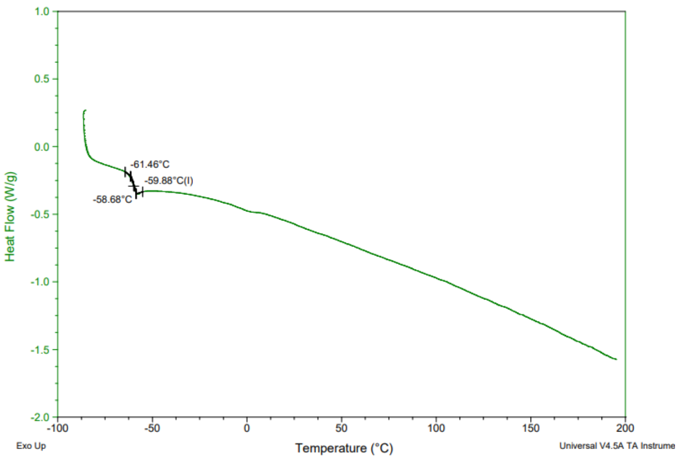

PEGA-Prop

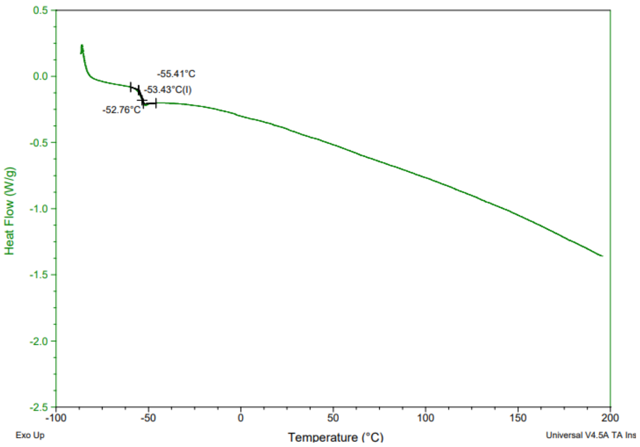

PEGA-SS

Table S1:Size and PDI values for the diol-modified pegylated polymers for a t=15 days.

**Polymer**	**Average size/ nm**	**PDI**
PEGA-Hex 50%	212.0 ± 1.5	0.12 ± 0.03
PEGA-Prop 50%	Peak 1: 163.1 ± 17.7<br>Peak 2: 1939 ± 654.4	0.41 ± 0.01
PEGA-Cyclo 50%	Peak 1: 368.3<br>Peak2: 108.2	0.33 ± 0.06
PEGA-BDM 50%	414.9 ± 1.3	0.13 ± 0.03
PEGA-SS 50%	269.4 ± 4.6	0.05 ± 0.02
